# A scoping review of global approaches to education in adult critical care retrieval

**DOI:** 10.1016/j.afjem.2026.100958

**Published:** 2026-02-26

**Authors:** Louis van Rensburg, Neville Vlok, Criag Vincent Lambert, Willem Stassen

**Affiliations:** a Division of Emergency Medicine, UCT, Cape Town, South Africa; b Emergency Medical Services, Western Cape Emergency Medical Services, Cape Town, South Africa; c Department of Emergency Medical Care, Faculty of Health Sciences, University of Johannesburg, South Africa

**Keywords:** Critical care, Patient transfer, Emergency medical services, Curriculum, South Africa

## Abstract

**Introduction:**

African healthcare systems remain under-resourced. As a consequence, critically ill or injured patients can find themselves in health care facilities that are unable to meet their complex needs. In such instances, referrals are made to larger, better-resourced facilities for ongoing care. Critical care retrieval (CCR) services are essential for ensuring timely and safe transfer of patients between healthcare facilities. In South Africa (SA), as in many settings, ambulance services are responsible for facilitating interfacility transfers. In high-income settings, CCR is recognised as a specialised area of practice. In African contexts, there is a growing recognition that current approaches to the education and training of EMS personnel to undertake CCR may be lacking. Through this scoping review we attempted to map both local and international CCR curricula as a basis for the development of a contextual CCR curriculum.

**Methods:**

An a priori search strategy was applied to identify peer-reviewed literature on adult CCRS training and curricula published between 2011 and August 2024. Databases searched included PubMed and Scopus, supplemented by Google and Google Scholar. Eligible studies were screened, mapped, and categorised into thematic domains aligned with a CCRS definition. Data extraction was conducted using a predesigned matrix, and findings were synthesised under emergent topic areas identified from the data.

**Results:**

Fifty-nine sources were included, with 85 % originating from high-income countries. Seven thematic domains were identified: the need for additional training and expanded scope of practice, call screening for specific patient populations, need for dedicated crew, equipment, quality management, and continuing medical education. There was also considerable variation in the focus, complexity and depth of training, with few standardised curriculum blueprints available that address CCR contexts seen in low- to middle-income settings such as SA.

**Conclusion:**

Findings highlight the need for a standardised, nationally recognised CCR curriculum in SA. Such a curriculum should integrate advanced clinical competencies, non-clinical skills, culturally sensitive education, and role of quality improvement processes. This scoping review provides a foundation for stakeholder-informed curriculum engagements and development to address these emergency care challenges which are common to many healthcare services.

## African Relevance

• Many African EMS systems lack standardised critical care retrieval training.
• Improved training may reduce the patient safety incidents and associated risks linked to interfacility transfers in African settings.
• Opportunity for Contextualised Curriculum Development in Africa.

## Introduction

Across Africa, low- to middle-income countries (LMICs) are experiencing an epidemiological transition from a historically high burden of communicable diseases to an increasing prevalence of non-communicable diseases (NCDs), largely driven by urbanisation, lifestyle change, and rising life expectancy [1]. The burden of NCDs in Sub-Saharan Africa has risen by an estimated 27 % over the past decade, while acute conditions such as trauma, maternal complications, and infectious diseases remain highly prevalent; together forming a “quadruple burden of disease” [2,3]

South Africa (SA) reflects this burden and faces additional system-level challenges including overcrowded facilities, inequitable resource distribution, and variable quality of care [3,4]. In this context, critically ill patients are often managed initially at facilities unable to provide definitive care and require transfer to better-resourced centres [5,6]. Interfacility transfers (IFTs) are therefore a vital component of the healthcare continuum and refer broadly to the movement of patients between healthcare facilities for access to appropriate levels of care. .Within this broader transfer function, Critical Care Retrieval (CCR) represents a specialised subset of transfers for critically ill or injured patients and has been formally defined and endorsed within the South African context as “*the stabilisation and transport of a critically ill or injured patient from a location where the patient’s healthcare requirements outweigh the diagnostic or treatment abilities, and/or expertise available, to an appropriate facility where these are available*” [7]. The process carries significant risk as patients are frequently physiologically unstable and vulnerable to deterioration during transport [5,6,8,9]. Evidence from high-income countries (HICs) demonstrates that adverse event rates can be substantially reduced when transfers are conducted by trained teams; one prospective study reported 22 % adverse events compared to 35–70 % in less structured settings [7](10).

To mitigate these risks, Emergency Medical Services (EMS) personnel require advanced training in CCR [11,12]. In HICs, CCR is typically managed by dedicated retrieval teams with structured curricula [12]. In SA, CCR is performed largely by Emergency Care Practitioners (ECPs) and paramedics trained via diverse educational routes, including the four-year Bachelor of Emergency Medical Care (BEMC) degree, national diplomas, two-year diplomas, and legacy short courses. These pathways result in inconsistent CCR preparation [5].

Despite the growing demand for CCR services, South Africa lacks a national training standard [5,7,10,11]. Providers have attempted to fill this gap with internal Continuing Professional Development (CPD) based initiatives, but these are fragmented, informal, and lack external validation. Operational exposure and undergraduate education alone have proven insufficient; structured, contextually relevant education is required to ensure competence and patient safety [5].

This scoping review aimed to map local and international CCR curricula to identify what is currently taught and what should be taught. By synthesising available evidence, we sought to provide a foundation for developing a standardised, evidence-informed curriculum tailored to Africa’s healthcare needs and resource constraints. Given that CCR is not yet well established in Africa, both undergraduate and postgraduate levels were considered, although internationally CCR training is most often positioned as a postgraduate specialisation.

## Methods

A scoping review methodology was appropriate given the variability in terminology, programme structure, and reporting across international adult CCR training initiatives [12]. The review, therefore, aimed to map available evidence, synthesise key domains, and identify gaps to support curriculum development. Accordingly, this scoping review explored adult CCR training programmes and curricula, both locally in South Africa and internationally, including grey literature and peer-reviewed sources published between January 2011 and August 2024.As the study relied exclusively on publicly available data, it was exempted from formal ethical review by the Human Research Ethics Committee of the University of Cape Town (HREC 408/2022). The review was conducted in accordance with the PRISMA-ScR guidelines [13].

Guidance from Aromataris et al. [14] was considered in developing the search strategy. Six thematic elements were deductively derived from the operational definition of CCR/CCRS and used as a priori domains for structured evidence mapping, consistent with established scoping review methodology(7,12). Six core thematic elements informed the strategy: (a) critical care retrieval, defined as interfacility transport of critically ill patients requiring specialised care; (b) CCR curricula, referring to structured content guiding knowledge and skills; (c) CCR training programmes designed to prepare healthcare professionals for critical care transport; (d) standards underpinning quality and safety; (e) learners, defined as providers undergoing CCR training; and (f) international experiences and models of CCR training.

Search strings combining these concepts with Boolean operators were applied in Medline (via PubMed) and Scopus. Because database searches yielded limited results, supplementary grey literature searches were performed using Google and Google Scholar with targeted keywords such as “critical care retrieval training programme” and “curricula.” Grey literature also included academic repositories, government sites, and professional platforms. Full database search strategies are available in Supplementary File 1.

Eligibility was restricted to English-language sources referencing CCR training or qualification frameworks in titles, keywords, or abstracts. Excluded sources were those outside the CCR domain, not in English, or without full-text access. After duplicate removal, two investigators (LvR, NV) independently screened titles and abstracts. Discrepancies were resolved during full-text review. Reference lists of included sources were also hand-searched.

Data were extracted from full texts into a predesigned Excel matrix (Microsoft Corp., Redmond, USA) capturing study aims, methods, sample characteristics, findings, limitations, and bibliographic details. The matrix was piloted independently by both reviewers, and domains were refined through consensus (Appendix 2). Disagreements were resolved through re-examination of full texts. No formal risk of bias assessment was performed, consistent with scoping review methodology [13].

Finally, the refined topic domains were aligned with a working definition of Critical Care Retrieval Services (CCRS) relevant to South Africa, characterised by patient populations and case selection, dedicated and specially trained crews, specialised equipment, and continuous quality assurance and training [7].

## Results

**Fig. 1:**
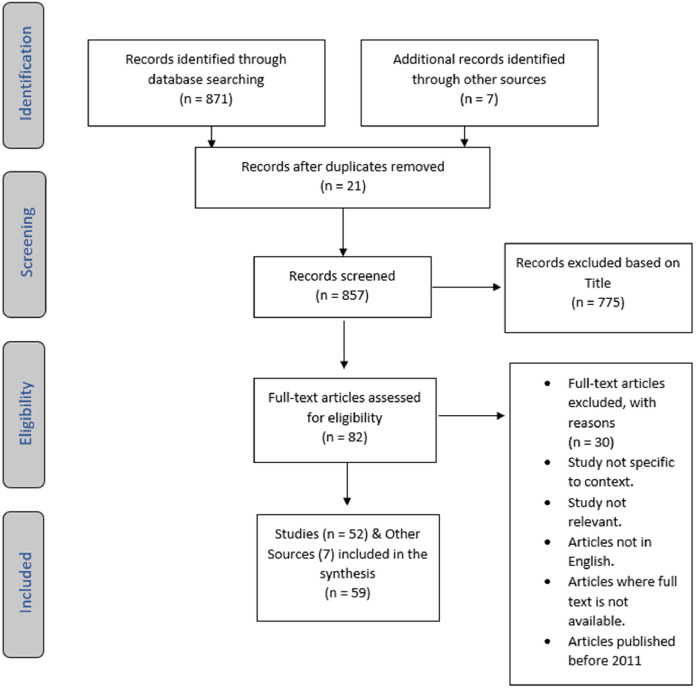
PRISMA flow diagram.

After screening 871 articles and removing duplicates, a total of 82 full-text articles were assessed, and 52 were included. An additional seven sources were identified through grey literature searches, resulting in 59 sources in total (Fig. 1).

**Fig. 2:**
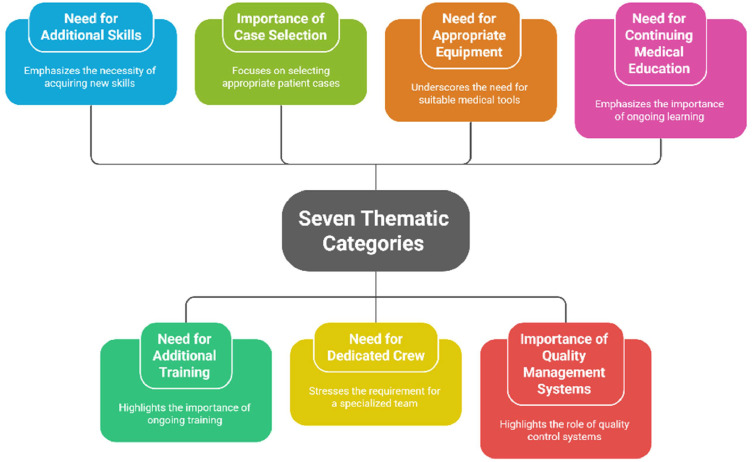
Seven thematic categories.

The majority of studies originated from HICs such as Australia, the United States, the United Kingdom, and Qatar, accounting for approximately 85 % of the total. In contrast, only 15 % originated from LMICs, including South Africa. All included literature was deductively mapped to a locally relevant framework for defining critical care retrieval services (CCRS), consisting of seven thematic categories as outlined in Fig. 2.

### Additional skills and expanded scope

This category included nine studies: six from the United States [[16], [17], [18], [19], [20], [21]], one each from Australia [22], the United Kingdom [23], and South Africa [10]. These studies used diverse methodologies, such as retrospective reviews, literature reviews, and Delphi processes. Across contexts, there is consensus that CCRS providers should possess an expanded clinical scope, including diagnostic and therapeutic skills tailored to high-acuity patients [10,[16], [17], [18],^22^,^23^]. In the South African context, some of these competencies may be additional to current HPCSA-defined scopes of practice, and their applicability may vary across practitioner categories involved in CCRS.

Several studies advocated for expanded competencies in advanced airway management and extracorporeal membrane oxygenation (ECMO), among others. Authors noted that these additions are safe and effective when accompanied by appropriate training and clinical governance [10,[19], [20], [21]]. These competencies are summarised in Table 1.

**Table 1:** Content derived from published literature.

Curriculum Content derived from published literature	
Teaching Additions	
**Category**	**Curriculum content (Teaching additions)**
**Airway & Ventilation**	**Advanced airway management; Rapid Sequence Intubation (RSI); Mechanical ventilation; Ultrasonography – Endotracheal Tube (ET) confirmation**
**Vascular Access & Invasive Procedures**	**Vascular access – Arterial stab; Vascular access – Central venous catheter; Arterial line insertion; Placement of chest drainage systems; Initiation of tracheostomy**
**Monitoring & Critical Care Devices**	**Arterial line monitoring; Invasive arterial pressure monitoring; Intracranial pressure monitoring; Monitoring of chest drainage systems; Monitoring of tracheostomy; Monitoring of transvenous pacing; Multi-channel infusion pump/syringe; Intra-aortic balloon pump monitoring; Extracorporeal membrane oxygenation; Pulmonary artery catheter removal**
**Diagnostics, Imaging & Point-of-Care Testing**	**Arterial Blood Gas analysis; Blood analysis of cardiac markers; Blood analysis of infection markers; Blood analysis of renal function; Cardiac ECG (12 lead); Chest X-ray interpretation; CT and MRI; Doppler ultrasound assessment; Ultrasonography – Lung**
**Medications, Infusions & Blood Products**	**ICU medication administration/management; Initiation and monitoring of blood product transfusion; Training specific to the equipment used in the ICU environment**
**Specialist Clinical Interventions**	**Balloon tamponade of gastroesophageal varices monitoring; REBOA; Obstetric balloon initiation; Obstetric balloon monitoring**
**Communication, Handover & Non-Technical Skills**	**Handover training; Non-technical skills; Content to be patient/caseload-centred**

### Additional training

Twenty-three studies highlighted the need for supplementary CCRS training. These included research from the USA (n=10) [[24], [25], [26], [27], [28], [29], [30], [31], [32], [33]], South Africa (n=3) [5,34,35], Australia (n=3) [[36], [37], [38]], the UK (n=2) [39,40], and individual studies from New Zealand [38], Canada [41], Japan [42], Norway [43], and Iran [44]. Methodologies ranged from simulation studies and curriculum analyses to surveys, trials, and interviews.

A dominant theme was that standard undergraduate EMS education does not adequately prepare providers for the CCR environment [5,10,34,44]. Studies also emphasised the importance of non-clinical skills—such as communication, teamwork, empathy, and structured handovers—as essential components of CCRS training [27,33,40,42,45]. Crew Resource Management (CRM) was cited as a method of imparting these competencies [35]. These findings are reflected in Table 2.

**Table 2:** Topics included in international critical care paramedic programmes.

Topics included in Critical Care Paramedic Programmes	
**Clinical Teaching Additions**	**Non-Clinical Skills**
**Additional diagnostic training**	**Effective communication and decision-making skills**
**Advanced airway training**	**Ethical and professional behaviour**
**Advanced Ventilation techniques**	**Factors that influence safe care**
**Flight physiology**	**Interprofessional teamwork**
**Geriatric and Special needs patients**	**Safe, effective, person-centred care**
**ICU Medications and infusion**	**Patient Advocacy**
**Intra-aortic balloon pump**	**How to practice evidence-based medicine**
**Special monitoring/assessment techniques (Ultrasonography, ABG Analysis, Invasive Haemodynamic monitoring)**	
**Transport and packaging considerations**	
**Transport radiology**	

### Case selection and specific patient populations

Five studies addressed this domain, including contributions from South Africa [46], the USA [47], Scotland [48], and Qatar [49]. Venter et al. reported on 1839 private-sector CCTs in South Africa, revealing cardiovascular disease (25 %), infections (10 %), trauma (21 %), and other complex diagnoses as leading causes for transfer [46].

Appropriate triage was emphasised to avoid CCRS dilution by low-acuity cases [47,[49], [50], [51]]. Studies from Scotland and South Africa highlighted the absence of a universal definition for “critically ill,” posing challenges in patient selection [7,15,48].

### Need for dedicated crews

Eight studies addressed the need for specialised crews, including those from the USA [[52], [53], [54]], UK [39,55], Canada [56], Sweden [57], and South Africa [5]. Findings showed that dedicated CCRS teams—rather than ad hoc assignments—are associated with improved outcomes and higher quality of care [53,55]. Crew compositions varied globally, with interdisciplinary models in the USA and physician-paramedic combinations elsewhere [49,52,57]. The literature pported professional specialisation and formal role delineation to enhance performance and safety [54].

### Need for appropriate equipment

Four studies explored equipment-related issues in CCRS, conducted in the USA [50,58], UK [51], and South Africa [5]. Dedicated ICU-level equipment was deemed essential for patient safety and clinical effectiveness [5,59]. South African practitioners reported being ill-equipped and underprepared for CCTs, reinforcing the need for standardised equipment and related training [5,51,60].

### Quality management systems

Five studies addressed continuous quality improvement (CQI) from the USA [[61], [62], [63], [64]] and Norway [65]. CQI practices—such as airway registries, case reviews, and benchmarking—were shown to improve care and reduce variability [[61], [62], [63],^66^]. Integrating QI training into CCRS education was recommended without significantly increasing programme complexity [63]. Benchmarking was also noted as crucial for driving quality, cost-efficiency, and patient safety [64].

### Continuing medical education

Ongoing professional development remains critical for CCRS practitioners. While most CME formats remain didactic, newer approaches—including high-fidelity simulation and blended web-based learning—are showing promise [[67], [68], [69]]. However, standardised CCRS-focused CME remains limited [33,35]. Studies have demonstrated that simulation and online platforms improve clinical skills, decision-making, and adherence to protocols [62,70,71].

## Discussion

This scoping review sought to explore the current landscape of adult CCR training programmes and curricula, both internationally and within South Africa, to inform the development of a context-specific curriculum. The main finding is that while a range of CCR training content exists, particularly in high-income countries, there is a marked absence of standardised, comprehensive, and contextually relevant curricula tailored to the realities of LMICS like South Africa.

### Interpreting the findings in context

The literature reviewed suggests that CCR is a highly specialised domain requiring advanced clinical, technical, and non-clinical competencies [49,72]. However, current training offerings particularly within South Africa are fragmented and inconsistently delivered [11,34]. Although many international programmes offer detailed curricula with clearly defined competencies, these are often shaped by high-resource environments and may not translate directly into settings where infrastructure, staffing, and resources are constrained.

The thematic domains used to present the findings reflect a framework for CCR services developed in the South African context [7]. These were not emergent themes but rather served as a deductive structure to categorise the data. While the literature aligned with this framework, the findings reveal deeper systemic issues: a lack of formal postgraduate CCR training, minimal interprofessional education, and gaps in the integration of quality improvement and non-clinical skills.

### Comparisons with existing literature

Previous South African studies have noted a mismatch between critical care content taught at the undergraduate level and the demands of real-world interfacility transfers [5,34] . This review confirms those concerns and extends them by showing that the problem is not unique to South Africa [72]. Even in better-resourced systems, there is recognition that general EMS training may fall short in preparing providers for the complexities of CCR [72].

Notably, while international programmes increasingly incorporate simulation, interdisciplinary team training, and outcome-focused quality assurance processes, these are either absent or inconsistently implemented in local programmes. This suggests a global trend toward the professionalisation and specialisation of CCR that South Africa has yet to fully adopt.

### Implications for South Africa and LMICs

In the South African context, where interfacility transfers are common due to systemic inequities a lack of structured CCR training carries significant risks [6,11]. These include suboptimal clinical care, increased adverse events, and inefficient use of limited EMS resources. A nationally endorsed curriculum could address these concerns by establishing a standard for practitioner readiness, ensuring equitable training access, and improving patient outcomes across both public and private sectors.

Moreover, the findings support the development of a CCR curriculum that is modular, flexible, and responsive to the realities of practice in LMICs. Such a programme should integrate non-clinical skills, clinical governance principles, and targeted training in equipment use, case selection, and team dynamics.

### Remaining gaps and future directions

This review underscores the urgent need for African-led research and curriculum development in CCR. The dominance of studies from high-income countries highlights a significant gap in the literature from LMICs, particularly South Africa. Moreover, there is limited published evidence on the effectiveness of current training in improving CCR outcomes locally an area that warrants focused attention.

Looking ahead, it is essential to bring together a wide range of stakeholders including clinicians, educators, policymakers, and community representatives to co-create a national CCRS curriculum that is both evidence-informed and contextually grounded. Piloting modular or blended learning models that incorporate simulation, digital platforms, and structured mentorship may offer a sustainable, scalable approach to developing a capable and confident CCR workforce.

Further research should prioritise evaluating the impact of such training on practitioner readiness and patient outcomes to ensure that educational innovations are both effective and aligned with the realities of emergency care delivery in South Africa and other LMICs. By addressing these gaps, this review lays the groundwork for a robust, adaptable curriculum that can raise the standard of care, reduce variability in clinical practice, and ultimately improve outcomes for critically ill and injured patients across the country.

## Limitations

This scoping review has several limitations. Although grey literature was included where available and applicable, some relevant local or unpublished training initiatives may not have been captured, potentially underrepresenting informal or emerging programmes. The predominance of sources from HICs (85 %) may limit the contextual relevance of some findings to LMIC settings such as South Africa. While grounded in a locally proposed definition of CCRS, it may have constrained the emergence of novel themes not explicitly aligned with it. As per scoping review methodology, no formal quality appraisal of included studies was performed, which restricts conclusions regarding the rigour of the underlying evidence. Lastly, the review focused solely on adult critical care retrieval and did not explore training relevant to paediatric or neonatal populations, which may have overlapping competencies.

## Conclusion

This scoping review identified a significant gap in standardised, contextually relevant CCR training programmes in Africa and other LMICs. While international models offer valuable insights, they are often grounded in high-resource contexts and are not easily transferable to settings with limited infrastructure and workforce variability. The findings highlight an urgent need for a nationally endorsed, modular CCR curriculum that reflects local operational realities and integrates clinical, non-clinical, and quality assurance competencies. Addressing this gap through stakeholder-driven curriculum development and pilot implementation could strengthen CCR capacity, improve patient outcomes, and build a more resilient and responsive regional emergency care system.

## Dissemination of results

Findings from this scoping review were shared with colleagues and stakeholders during a dedicated Western Cape EMS Research Day.

## Credit author statement

L.v.R.: Conceptualisation, Methodology, Investigation, Formal analysis, Project administration, Software, Validation, Writing – original draft, Writing – review and editing. N.V.: Formal analysis, Writing – original draft, Writing – review and editing. C.V-L.: Supervision, Writing – review and editing. W.S.: Conceptualisation, Methodology, Data curation, Project administration, Resources, Supervision, Writing – review and editing.

C.V-L. and W.S. also served as academic supervisors on this study.

All authors approved the version to be published and agreed to be accountable for all aspects of the work

## Declaration of competing interest

WS and CVL are editors of the journal, but were not involved in the editorial processes and decisions aroudn this submission. The authors declare that they have no known competing financial interests or personal relationships that could have appeared to influence the work reported in this paper.

## Appendix Supplementary materials

Image, application 1

Image, application 2
