# Supplementary material for: A scoping review of global approaches to education in adult critical care retrieval
Source: Afr J Emerg Med. 2026 Feb 26;16(2):100958. doi: 10.1016/j.afjem.2026.100958 (PMC12962074; doi:10.1016/j.afjem.2026.100958)
Supplement: Supplementary file 2 [file mmc2.docx]

**Appendix 1: Search Strategy**

Database: PUBMED:

Search date: 30/09/2022

Number of results: 713 (REPEATED 13/08/2024)

(((((((((((Critical Care Retrieval) OR (Critical Care Transport)) OR (Critical Care Transfer)) AND (y_10[Filter])) AND ((((Curriculum)) OR (Syllabus)) OR (Educational Framework) AND (y_10[Filter]))) AND (((Training) OR (Education)) OR (Learning) AND (y_10[Filter]))) AND (((Standards) OR (Criteria)) OR (Requirements) AND (y_10[Filter])),,in the last 10 years,"((((""critical care""[MeSH Terms] OR (""critical""[All Fields] AND ""care""[All Fields]) OR ""critical care""[All Fields]) AND (""retrievability""[All Fields] OR ""retrievable""[All Fields] OR ""retrieval""[All Fields] OR ""retrievals""[All Fields] OR ""retrieve""[All Fields] OR ""retrieved""[All Fields] OR ""retrieves""[All Fields] OR ""retrieving""[All Fields])) OR ((""critical care""[MeSH Terms] OR (""critical""[All Fields] AND ""care""[All Fields]) OR ""critical care""[All Fields]) AND (""biological transport""[MeSH Terms] OR (""biological""[All Fields] AND ""transport""[All Fields]) OR ""biological transport""[All Fields] OR ""transport""[All Fields] OR ""membrane transport proteins""[MeSH Terms] OR (""membrane""[All Fields] AND ""transport""[All Fields] AND ""proteins""[All Fields]) OR ""membrane transport proteins""[All Fields] OR ""transporter""[All Fields] OR ""transporters""[All Fields] OR ""transportable""[All Fields] OR ""transportation""[MeSH Terms] OR ""transportation""[All Fields] OR ""transportations""[All Fields] OR ""transported""[All Fields] OR ""transporter s""[All Fields] OR ""transporting""[All Fields] OR ""transports""[All Fields])) OR ((""critical care""[MeSH Terms] OR (""critical""[All Fields] AND ""care""[All Fields]) OR ""critical care""[All Fields]) AND (""transfer""[All Fields] OR ""transferability""[All Fields] OR ""transferable""[All Fields] OR ""transfered""[All Fields] OR ""transfering""[All Fields] OR ""transferred""[All Fields] OR ""transferring""[All Fields] OR ""transfers""[All Fields]))) AND ""2012/09/01 00:00"":""3000/01/01 05:00""[Date - Publication] AND ((""curriculum""[MeSH Terms] OR ""curriculum""[All Fields] OR ""curricula""[All Fields] OR ""curriculums""[All Fields] OR ""curriculum s""[All Fields] OR ""education""[MeSH Subheading] OR ""education""[All Fields] OR ""Syllabus""[All Fields] OR ((""educability""[All Fields] OR ""educable""[All Fields] OR ""educates""[All Fields] OR ""education""[MeSH Subheading] OR ""education""[All Fields] OR ""educational status""[MeSH Terms] OR (""educational""[All Fields] AND ""status""[All Fields]) OR ""educational status""[All Fields] OR ""education""[MeSH Terms] OR ""education s""[All Fields] OR ""educational""[All Fields] OR ""educative""[All Fields] OR ""educator""[All Fields] OR ""educator s""[All Fields] OR ""educators""[All Fields] OR ""teaching""[MeSH Terms] OR ""teaching""[All Fields] OR ""educate""[All Fields] OR ""educated""[All Fields] OR ""educating""[All Fields] OR ""educations""[All Fields]) AND (""framework""[All Fields] OR ""framework s""[All Fields] OR ""frameworks""[All Fields]))) AND ""2012/09/01 00:00"":""3000/01/01 05:00""[Date - Publication]) AND ((""education""[MeSH Subheading] OR ""education""[All Fields] OR ""training""[All Fields] OR ""education""[MeSH Terms] OR ""train""[All Fields] OR ""train s""[All Fields] OR ""trained""[All Fields] OR ""training s""[All Fields] OR ""trainings""[All Fields] OR ""trains""[All Fields] OR (""educability""[All Fields] OR ""educable""[All Fields] OR ""educates""[All Fields] OR ""education""[MeSH Subheading] OR ""education""[All Fields] OR ""educational status""[MeSH Terms] OR (""educational""[All Fields] AND ""status""[All Fields]) OR ""educational status""[All Fields] OR ""education""[MeSH Terms] OR ""education s""[All Fields] OR ""educational""[All Fields] OR ""educative""[All Fields] OR ""educator""[All Fields] OR ""educator s""[All Fields] OR ""educators""[All Fields] OR ""teaching""[MeSH Terms] OR ""teaching""[All Fields] OR ""educate""[All Fields] OR ""educated""[All Fields] OR ""educating""[All Fields] OR ""educations""[All Fields]) OR (""learning""[MeSH Terms] OR ""learning""[All Fields] OR ""learn""[All Fields] OR ""learned""[All Fields] OR ""learning s""[All Fields] OR ""learnings""[All Fields] OR ""learns""[All Fields])) AND ""2012/09/01 00:00"":""3000/01/01 05:00""[Date - Publication]) AND ((""reference standards""[MeSH Terms] OR (""reference""[All Fields] AND ""standards""[All Fields]) OR ""reference standards""[All Fields] OR ""standardization""[All Fields] OR ""standard""[All Fields] OR ""standard s""[All Fields] OR ""standardisation""[All Fields] OR ""standardisations""[All Fields] OR ""standardise""[All Fields] OR ""standardised""[All Fields] OR ""standardises""[All Fields] OR ""standardising""[All Fields] OR ""standardization s""[All Fields] OR ""standardizations""[All Fields] OR ""standardize""[All Fields] OR ""standardized""[All Fields] OR ""standardizes""[All Fields] OR ""standardizing""[All Fields] OR ""standards""[MeSH Subheading] OR ""standards""[All Fields] OR (""criteria s""[All Fields] OR ""criterias""[All Fields] OR ""standards""[MeSH Subheading] OR ""standards""[All Fields] OR ""criteria""[All Fields]) OR (""require""[All Fields] OR ""required""[All Fields] OR ""requirement""[All Fields] OR ""requirements""[All Fields] OR ""requires""[All Fields] OR ""requiring""[All Fields])) AND ""2012/09/01 00:00"":""3000/01/01 05:00""[Date - Publication])) AND (y_10[Filter])",713,07:46:46

((Standards) OR (Criteria)) OR (Requirements),,in the last 10 years,"(""reference standards""[MeSH Terms] OR (""reference""[All Fields] AND ""standards""[All Fields]) OR ""reference standards""[All Fields] OR ""standardization""[All Fields] OR ""standard""[All Fields] OR ""standard s""[All Fields] OR ""standardisation""[All Fields] OR ""standardisations""[All Fields] OR ""standardise""[All Fields] OR ""standardised""[All Fields] OR ""standardises""[All Fields] OR ""standardising""[All Fields] OR ""standardization s""[All Fields] OR ""standardizations""[All Fields] OR ""standardize""[All Fields] OR ""standardized""[All Fields] OR ""standardizes""[All Fields] OR ""standardizing""[All Fields] OR ""standards""[MeSH Subheading] OR ""standards""[All Fields] OR (""criteria s""[All Fields] OR ""criterias""[All Fields] OR ""standards""[MeSH Subheading] OR ""standards""[All Fields] OR ""criteria""[All Fields]) OR (""require""[All Fields] OR ""required""[All Fields] OR ""requirement""[All Fields] OR ""requirements""[All Fields] OR ""requires""[All Fields] OR ""requiring""[All Fields])) AND (y_10[Filter])","2,231,595",07:44:37

((Training) OR (Education)) OR (Learning),,in the last 10 years,"(""education""[MeSH Subheading] OR ""education""[All Fields] OR ""training""[All Fields] OR ""education""[MeSH Terms] OR ""train""[All Fields] OR ""train s""[All Fields] OR ""trained""[All Fields] OR ""training s""[All Fields] OR ""trainings""[All Fields] OR ""trains""[All Fields] OR (""educability""[All Fields] OR ""educable""[All Fields] OR ""educates""[All Fields] OR ""education""[MeSH Subheading] OR ""education""[All Fields] OR ""educational status""[MeSH Terms] OR (""educational""[All Fields] AND ""status""[All Fields]) OR ""educational status""[All Fields] OR ""education""[MeSH Terms] OR ""education s""[All Fields] OR ""educational""[All Fields] OR ""educative""[All Fields] OR ""educator""[All Fields] OR ""educator s""[All Fields] OR ""educators""[All Fields] OR ""teaching""[MeSH Terms] OR ""teaching""[All Fields] OR ""educate""[All Fields] OR ""educated""[All Fields] OR ""educating""[All Fields] OR ""educations""[All Fields]) OR (""learning""[MeSH Terms] OR ""learning""[All Fields] OR ""learn""[All Fields] OR ""learned""[All Fields] OR ""learning s""[All Fields] OR ""learnings""[All Fields] OR ""learns""[All Fields])) AND (y_10[Filter])","1,729,804",07:42:20

(((Curriculum)) OR (Syllabus)) OR (Educational Framework),,in the last 10 years,"(""curriculum""[MeSH Terms] OR ""curriculum""[All Fields] OR ""curricula""[All Fields] OR ""curriculums""[All Fields] OR ""curriculum s""[All Fields] OR ""education""[MeSH Subheading] OR ""education""[All Fields] OR ""Syllabus""[All Fields] OR ((""educability""[All Fields] OR ""educable""[All Fields] OR ""educates""[All Fields] OR ""education""[MeSH Subheading] OR ""education""[All Fields] OR ""educational status""[MeSH Terms] OR (""educational""[All Fields] AND ""status""[All Fields]) OR ""educational status""[All Fields] OR ""education""[MeSH Terms] OR ""education s""[All Fields] OR ""educational""[All Fields] OR ""educative""[All Fields] OR ""educator""[All Fields] OR ""educator s""[All Fields] OR ""educators""[All Fields] OR ""teaching""[MeSH Terms] OR ""teaching""[All Fields] OR ""educate""[All Fields] OR ""educated""[All Fields] OR ""educating""[All Fields] OR ""educations""[All Fields]) AND (""framework""[All Fields] OR ""framework s""[All Fields] OR ""frameworks""[All Fields]))) AND (y_10[Filter])","893,695",07:40:54

((((((((Critical Care Retrieval) OR (Critical Care Transport)) OR (Critical Care Transfer)),,in the last 10 years,"(((""critical care""[MeSH Terms] OR (""critical""[All Fields] AND ""care""[All Fields]) OR ""critical care""[All Fields]) AND (""retrievability""[All Fields] OR ""retrievable""[All Fields] OR ""retrieval""[All Fields] OR ""retrievals""[All Fields] OR ""retrieve""[All Fields] OR ""retrieved""[All Fields] OR ""retrieves""[All Fields] OR ""retrieving""[All Fields])) OR ((""critical care""[MeSH Terms] OR (""critical""[All Fields] AND ""care""[All Fields]) OR ""critical care""[All Fields]) AND (""biological transport""[MeSH Terms] OR (""biological""[All Fields] AND ""transport""[All Fields]) OR ""biological transport""[All Fields] OR ""transport""[All Fields] OR ""membrane transport proteins""[MeSH Terms] OR (""membrane""[All Fields] AND ""transport""[All Fields] AND ""proteins""[All Fields]) OR ""membrane transport proteins""[All Fields] OR ""transporter""[All Fields] OR ""transporters""[All Fields] OR ""transportable""[All Fields] OR ""transportation""[MeSH Terms] OR ""transportation""[All Fields] OR ""transportations""[All Fields] OR ""transported""[All Fields] OR ""transporter s""[All Fields] OR ""transporting""[All Fields] OR ""transports""[All Fields])) OR ((""critical care""[MeSH Terms] OR (""critical""[All Fields] AND ""care""[All Fields]) OR ""critical care""[All Fields]) AND (""transfer""[All Fields] OR ""transferability""[All Fields] OR ""transferable""[All Fields] OR ""transfered""[All Fields] OR ""transfering""[All Fields] OR ""transferred""[All Fields] OR ""transferring""[All Fields] OR ""transfers""[All Fields]))) AND (y_10[Filter])","13,927",07:36:07

Database: Scopus:

Search date: 30/09/2022

Number of results: 157 (REPEATED 13/08/2024)

Critical Care Retrieval OR Critical Care Transport OR Critical Care Transfer AND Curriculum OR Syllabus OR Educational Framework AND Training OR Education OR Learning AND standards OR criteria OR requirements AND ( LIMIT-TO ( SUBJAREA,"MEDI" ) OR LIMIT-TO ( SUBJAREA,"NURS" ) OR LIMIT-TO ( SUBJAREA,"HEAL" ) OR LIMIT-TO ( SUBJAREA,"PHAR" ) OR LIMIT-TO ( SUBJAREA,"MULT" ) ) AND ( LIMIT-TO ( PUBYEAR,2023) OR LIMIT-TO ( PUBYEAR,2022) OR LIMIT-TO ( PUBYEAR,2021) OR LIMIT-TO ( PUBYEAR,2020) OR LIMIT-TO ( PUBYEAR,2019) OR LIMIT-TO ( PUBYEAR,2018) OR LIMIT-TO ( PUBYEAR,2017) OR LIMIT-TO ( PUBYEAR,2016) OR LIMIT-TO ( PUBYEAR,2015) OR LIMIT-TO ( PUBYEAR,2014) OR LIMIT-TO ( PUBYEAR,2013) OR LIMIT-TO ( PUBYEAR,2012) )
